# Biochemically Silent Pheochromocytoma in an Asymptomatic Patient With a Unilateral Lipid-Poor Adrenal Adenoma

**DOI:** 10.7759/cureus.47120

**Published:** 2023-10-16

**Authors:** Akbar Hussain, Sanjana Thota Kammili, Edilfavia Uy, Jonathan Piercy, Shyam Ganti, Dedeepya Gullapalli

**Affiliations:** 1 Internal Medicine, Appalachian Regional Healthcare, Harlan, USA; 2 Internal Medicine, Appalachian Regional Healthcare, Whitesburg, USA; 3 Diabetes and Endocrinology, Appalachian Regional Healthcare, Whitesburg, USA

**Keywords:** adrenal medulla, chromaffin cells, pheochromocytoma, incidentaloma, catecholamine

## Abstract

In this case, a Caucasian woman was incidentally found to have a left adrenal gland incidentaloma a decade ago. Initial tests indicated a non-functional lipid-poor adenoma, but ongoing surveillance revealed irregularities in biochemical testing for pheochromocytoma. The patient was concurrently taking an SNRI, known to elevate biochemical markers artificially. Given the adenoma’s growth and mild biochemical abnormalities, laparoscopic surgery was performed, and the tumor was found to be a 2.4 cm × 1.8 cm pheochromocytoma. Following the procedure, hormone levels normalized, and the patient experienced relief from symptoms. This case underscores the rarity of pheochromocytomas, emphasizing the importance of accurate diagnosis and effective management. Imaging techniques, notably computed tomography (CT) and magnetic resonance imaging (MRI), played a crucial role in localization, particularly through contrast-enhanced methods. Key characteristics like Hounsfield density, enhancement patterns, and washout behavior aided in distinguishing diverse adrenal masses. For cases where imaging had limitations, complementary techniques such as 23I-metaiodobenzylguanidine (MIBG) scintigraphy, specialized MR sequences, and GA-DOTATATE scans provided supplementary diagnostic insights, collectively contributing to a comprehensive clinical understanding. Despite advancements, challenges persist in differentiating specific adrenal tumors, highlighting the need for continued research and refined imaging methodologies.

## Introduction

Pheochromocytomas are rare catecholamine-secreting tumors arising from the adrenal medulla that can occur in asymptomatic patients. About 5.0-6.5% of adrenal incidentalomas are pheochromocytomas, with approximately 8% of these cases being asymptomatic and often familial [1]. Chromaffin cells in the sympathoadrenal system give rise to catecholamine-producing tumors [2]. Catecholamine tumors are relatively common among hypertensive outpatients, occurring in 0.2-0.6% of cases [3]. Eighty percent of the hormone produced by the adrenal medulla is epinephrine; however, tumors arising from the medulla have a higher proportion of norepinephrine compared to normal medullary tissue [4].

Elevated catecholamines lead to symptoms like headaches, rapid heartbeats, sweating, anxiety, and breathing difficulties. They can also impact insulin, causing high blood glucose, and in addition, they can lead to severe complications like heart disease and brain hemorrhage. Diagnostic assessment often involves measuring blood or urine metanephrine levels to detect catecholamine overproduction [5]. However, in rare cases, catecholamine levels are within normal limits. These are known as “biochemically silent” pheochromocytomas. The lack of biochemical evidence makes diagnosis particularly challenging in cases presenting as adrenal incidentalomas, which can only be found by chance during radiographic examinations of the adrenal glands.

We present a patient with a left adrenal incidentaloma, initially identified 10 years ago, that has been slowly growing and gradually becoming biochemically active. This case illustrates how radiologic findings can alert providers to the presence of pheochromocytoma and the importance of not immediately attributing mild biochemical abnormalities to medications. In addition, it is noteworthy that this case report was previously presented as a poster abstract at the Endocrine Society Conference held in Atlanta in June 2022.

## Case presentation

We present a case of a 50-year-old Caucasian female who presented to the clinic for evaluation of a left adrenal incidentaloma discovered 10 years ago. Past medical history is significant for hypothyroidism, dyslipidemia, anxiety, and depression. The patient had undergone surveillance of the adrenal nodule, both radiographically and biochemically, since its discovery.

**Figure 1:**
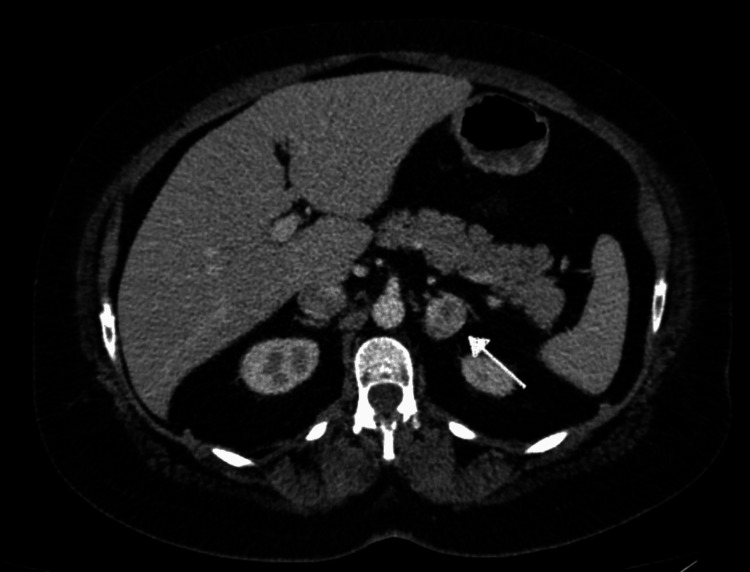
CT abdomen and pelvis revealing a 2.6 cm left adrenal nodule (highlighted by white arrow)

The first available image is a computed tomography (CT) of the abdomen and pelvis five years back, which noted a 1.7 cm left adrenal nodule with an unenhanced attenuation value of 25.6 HU. The most recent CT of the abdomen and pelvis reported a 2.6 cm mixed density enhancing the left adrenal gland nodule with less apparent macroscopic fat, concerning a lipid-poor adenoma, as shown in Figure 1.

The initial biochemical assessment revealed elevated 24-hour urine normetanephrine at 859 mcg/24h (reference range: 88-649 mcg/24h) and elevated 24-hour total urine metanephrines at 955 mcg/24h (reference range: 182-739 mcg/24h). Despite these findings, no immediate intervention was pursued. However, during a recent visit, a subsequent evaluation showed elevated plasma normetanephrine at 308.6 pg/mL (reference range: 0-136.8 pg/mL), along with elevated 24-hour urine norepinephrine at 165 mcg/24h (reference range: 0-135 mcg/24h) and elevated urine 24-hour normetanephrine at 1015 mcg/24h (reference range: 131-612 mcg/24h), as summarized in Table 1.

**Table 1:** Comparison of biochemical parameters between initial and recent visits

Biochemical parameters	Normal limit	Initial values	Recent values
24-h urine metanephrines	Less than 350 µg/24h	99 ug/24h	154 ug/24h
24-h urine normetanephrine	Less than 600 µg/24h	859 ug/24h	1015 ug/24h
24-h urine epinephrine	Less than 20 µg/24h	<3 ug/24h	11 ug/24h
24-h urine norepinephrine	Less than 90 µg/24h	62 ug/24h	165 ug/24h
24-h urine dopamine	Less than 400 µg/24h	172 ug/24h	425 ug/24h
24-h urine vanillylmandelic acid (VMA)	Less than 7.7 mg/24h	2.4 mg/24h	NR

Aside from these mildly elevated values, the remainder of her biochemical workup was unremarkable. It was noted that the patient was concurrently taking Effexor, which has been known to cause falsely elevated normetanephrine levels. Nonetheless, given the progressing size of the lipid-poor adenoma, the patient was directed for an endocrine surgery evaluation. Preoperatively, she was started on doxazosin and subsequently underwent successful laparoscopic surgical removal, with pathology confirming a 2.4 cm pheochromocytoma. Post-surgery, plasma metanephrines and normetanephrines normalized. Genetic testing revealed no syndromic mutation.

## Discussion

Pheochromocytomas are rare tumors of the adrenal medulla that produce abundant catecholamines from chromaffin tissue. There are approximately two to eight cases of pheochromocytoma per million people [6], but they are sometimes associated with significant morbidity due to the physiological effects of catecholamines. In particular, hypertension related to pheochromocytoma can be fatal if left untreated. A hypertensive crisis or shock can occur even in previously asymptomatic patients who are receiving medications, undergoing surgery, or pregnant [7].

The increasing use of radiological imaging has led to the more frequent discovery of adrenal incidentalomas in older adults. Diagnosis of these findings depends heavily on the functional status and aggressiveness of the tumor. It is critical for functional assessment that cortisol, androgens, catecholamines, and aldosterone are being measured [8].

Silent pheochromocytomas, which exhibit minimal hormone elevation or are entirely asymptomatic, pose a significant diagnostic challenge and potentially life-threatening complications. They always lack overt symptoms, by definition, and may produce only a mild elevation in hormone levels, often below traditional diagnostic thresholds. This necessitates a nuanced diagnostic approach that goes beyond typical hormone thresholds, as undiagnosed pheochromocytomas can lead to hypertensive crises, cardiovascular events, and even death.

Factors influencing hormone levels and potentially masking silent pheochromocytomas include medications like beta-blockers and alpha-methyltyrosine, fluctuations in hormonal secretion, and the intermittent nature of some pheochromocytomas. Careful patient history-taking and understanding the impact of medications are vital components of accurate diagnosis.

Pheochromocytomas are typically located using magnetic resonance imaging (MRI) and a contrast-enhanced CT scan [9]. Due to the excellent spatial resolution in the pelvis, thorax, and abdomen, the Endocrine Society Guidelines recommend CT rather than MRI as the initial imaging modality for the majority of patients [3].

Imaging findings that point to adrenocortical carcinoma, metastasis, or pheochromocytoma include heterogeneity, hemorrhagic changes, high Hounsfield density (>10 HU) on CT, marked enhancement with intravenous contrast, delayed contrast washout (less than 60% within 10 min), or high signal intensity on T2-weighted MRI. In delayed CT scanning, a pheochromocytoma with lipid degeneration might produce low attenuation scores (10 HU) and more than 60% washout, which can resemble adrenal adenomas [10]. Size less than 5 cm, lack of appreciable growth on serial imaging, crisp margins, low attenuation scores, and more than 60% washout by delayed CT are characteristics of benign adrenal adenomas [11].

Pheochromocytoma is localized using contrast-enhanced imaging on MRI and CT scans [12]. To rule out extra-adrenal paraganglioma or metastases of very big tumors, metaiodobenzylguanidine (MIBG) and positron emission tomography (PET) scans are typically utilized. In a PET test, it was discovered that gallium DOTA-TOC/NOC and DOPA-PET performed better than FDG-PET at detecting paragangliomas. High Hounsfield density (10 HU) on CT scans, pronounced contrast enhancement, delayed washout of contrast agent (60% at 10 min), and high signal intensity and cystic T2-weighted MRI on T2-weighted MRI are all characteristics of heterogeneity or hemorrhagic alterations that may indicate metastasis, pheochromocytoma, adrenocortical cancer, etc. However, symptomatic individuals’ CT results revealed a tendency toward more calcification. Attenuation values of 10 HU, sharp edges, smooth contours, absence of visible growth on screening, and >60% washout on delayed CT scans are the characteristics of benign adrenal incidental tumors [11].

Growths unseen by X-ray or CT might be found with 23I-MIBG scintigraphy. It can likewise be used to distinguish other related masses elsewhere in the body, such as metastases, multifocal lesions, or paragangliomas, which emit catecholamines [13, 14]. An assortment of elective MR successions and techniques have been tried in later examinations to separate adrenal adenomas from other adrenal masses. Compound shift imaging is the most promising of these latter methods. It has been shown to be 96-100% accurate at telling the difference between adrenal adenomas and metastases [15].

The extent of overlap suggests that, despite the assistance of fat-suppressed imaging and gadolinium enhancement in reliably differentiating groups of arbitrarily designated adrenal tumors, it is still challenging to categorize particular patients. But another accomplishment of midfield MRI seems to be post-contrast adrenal tissue examination using a modified three-point Dixon approach.

While not the typical presentation, it’s crucial to consider that adrenal incidentalomas, particularly lipid-poor adenomas, can initially remain biochemically silent, not overproducing hormones. Over time, these nodules may exhibit mild hormonal activity or grow in size, eventually increasing hormone secretion. Consequently, even when not biochemically active, lipid-poor adenomas should be closely monitored or potentially removed due to the risk of developing into pheochromocytomas, cancer, or other conditions. This case serves as a valuable learning point. Additionally, it’s important to note that the patient takes venlafaxine, which can sometimes lead to false positive laboratory results. Knowledge of medications causing falsely elevated catecholamine levels is necessary; however, these should be considered along with other clinical and radiological findings. Nonetheless, given the adrenal nodule’s phenotype, concerns about the possibility of pheochromocytoma should still be addressed.

Phenotypic characteristics should take precedence over biochemical lab results when dealing with silent cases. Pathological examinations of lipid-poor adrenal nodules have shown that they can indeed turn out to be pheochromocytomas, even when preoperative biochemical testing appears normal. This underscores the importance of vigilance. While the more than two-fold cut-off in hormone levels may seem arbitrary, it reflects the common presentation of pheochromocytomas when detected in most cases [16]. However, it’s essential to remember that pheochromocytomas can remain biochemically silent initially, emphasizing the need for a comprehensive evaluation.

Surgical intervention is crucial for definitive pheochromocytoma diagnosis, and it should not always be ruled out due to the absence of overt symptoms or significant biochemical marker elevations. Untreated pheochromocytomas pose substantial risks during medical interventions, including surgery and anesthesia administration. Current guidelines suggest surgical removal considering tumor size, characteristics, and potential complications. A personalized decision-making process integrating multiple factors is crucial for optimal patient outcomes.

## Conclusions

Pheochromocytoma must be identified right away since delaying treatment might cause serious morbidity and death. In the early years after our patient’s adrenal adenoma was discovered, biochemical testing revealed normal results, and the patient was asymptomatic. A CT scan continued to show a lipid-poor adenoma with continued growth. Later biochemical testing was concerned for pheochromocytoma, which was confirmed after laparoscopic adrenalectomy. This case emphasizes the importance of surveillance for a lipid-poor adrenal nodule. Providers should have a high level of suspicion for pheochromocytoma in patients with lipid-poor adenomas, even if they are biochemically silent.
